# Personalization of Training and Weight Reduction Using Artificial Intelligence: A Scoping Review of Current Evidence and Practical Limitations

**DOI:** 10.3390/jfmk11030347

**Published:** 2026-08-31

**Authors:** Nebojša Čokorilo, Aleksa Čović, Branislav Kokeza, Marko Sadojević, Filip Marković

**Affiliations:** Faculty of Sport and Physical Education, University of Novi Sad, 21000 Novi Sad, Serbia; branislavkokeza7@gmail.com (B.K.); markosadojevic00@gmail.com (M.S.); ficamarkovic5566@gmail.com (F.M.)

**Keywords:** artificial intelligence, personalized intervention, exercise, physical activity, weight management, digital health, behavioral adherence

## Abstract

**Background and Objectives:** Artificial intelligence (AI) has rapidly emerged as a promising tool for delivering personalized interventions in physical activity, exercise prescription, and weight management. AI technologies may facilitate individualized recommendations, behavioral support, and lifestyle modification through adaptive digital health solutions, although their effectiveness remains to be established across different populations and settings. However, the current evidence remains heterogeneous, and the practical implementation of AI in personalized training and weight management requires further evaluation. This scoping review aimed to summarize the current evidence regarding the application of artificial intelligence for the personalization of training and weight reduction, with particular emphasis on the types of AI technologies used, their reported outcomes, practical applications, and current limitations. **Methods:** A scoping review was conducted following a structured literature search of studies investigating AI-supported interventions related to physical activity, exercise, dietary behavior, and weight management. Eight studies involving diverse populations, intervention designs, and AI technologies were included. Data were extracted on study characteristics, AI technologies, intervention characteristics, reported outcomes, and research gaps. The literature search was subject to access-based restrictions, including the use of “Free full text” in PubMed and “Open Access” in Web of Science, as well as language restrictions. **Results:** The included studies investigated a wide range of AI technologies, including conversational chatbots, natural language processing systems, machine learning algorithms, computer vision applications, knowledge-based systems, and large language models. Selected studies reported favorable or modest changes in exercise adherence, physical activity participation, dietary behaviors, user engagement, and weight-related outcomes; however, the magnitude and consistency of these findings varied across studies. Personalized coaching, real-time feedback, and continuous behavioral support were common features of interventions reporting favorable outcomes. However, considerable heterogeneity existed across study designs, participant populations, intervention protocols, AI technologies, and outcome measures, and evidence regarding long-term effectiveness remains limited. The findings should be interpreted in the context of the adopted search strategy, including access-based restrictions and the inability to retrieve eight of 57 reports sought for retrieval, which may have contributed to availability bias. **Conclusions:** Current evidence suggests that artificial intelligence may have potential as a tool for supporting the personalization of training and weight management interventions, particularly through individualized behavioral support, feedback, and user engagement. However, the available evidence is heterogeneous and does not yet allow firm conclusions regarding the effectiveness or mechanisms of AI-supported interventions. AI should currently be viewed as a complement rather than a replacement for healthcare and exercise professionals. Future large-scale randomized controlled trials with longer follow-up periods and standardized outcome measures are needed to clarify the effectiveness, sustainability, and practical implementation of AI-supported interventions.

## 1. Introduction

Artificial intelligence (AI) is increasingly being integrated into health-related applications, including physical activity and nutrition interventions, with the potential to support personalization, adherence, and health-related outcomes [1,2,3,4]. Traditional exercise and nutritional programs frequently suffer from poor long-term compliance because adherence is influenced not only by program design but also by motivational and psychological factors such as autonomy, competence, and perceived social support [5,6,7,8]. In addition, conventional approaches often have limited capacity to accurately estimate energy expenditure and deliver individualized exercise and dietary prescriptions, whereas recent technological advances—including AI, wearable technologies, mobile health applications, and machine-learning algorithms—can facilitate continuous monitoring of user behavior and physiological responses and may support more adaptive and personalized exercise and nutritional guidance [1,2,3,4,9,10,11].

Artificial intelligence has become increasingly relevant in obesity prevention and weight management, which remain major global public health challenges because excess body weight substantially increases the risk of cardiovascular disease, type 2 diabetes, musculoskeletal disorders, and reduced quality of life [1,2,12,13]. Although conventional weight-reduction strategies can achieve short-term success, long-term maintenance remains difficult due to poor adherence and insufficient personalization of interventions [14]. AI-supported systems may address these limitations by providing real-time feedback, automated progress monitoring, personalized goal setting, continuous behavioral guidance, and improved accessibility to individualized exercise and nutrition support outside traditional clinical settings [1,2,15,16]. Nevertheless, concerns regarding data privacy, algorithm transparency, safety, and the reliability of automatically generated recommendations continue to limit widespread implementation [4,10,17,18].

Existing studies differ substantially in methodology, population characteristics, intervention types, and outcomes measured, making it difficult to clearly determine the overall effectiveness and practical applicability of AI-supported interventions in kinesiology and nutrition [1,2]. Moreover, the rapid development of AI technologies has resulted in a growing body of heterogeneous evidence that has not yet been comprehensively mapped [1,2]. The review question was formulated according to the Population–Concept–Context (PCC) framework recommended for scoping reviews [19]. The review sought to answer the following question: What is the current evidence regarding the use of artificial intelligence to personalize exercise, physical activity, nutrition, rehabilitation, and behavioral interventions that support training optimization and weight management? The PCC framework was defined as follows:

- Population (P): adolescents and adults participating in AI-supported lifestyle interventions.
- Concept (C): application of artificial intelligence to personalize exercise prescription, physical activity promotion, nutritional guidance, behavioral support, and rehabilitation strategies aimed at improving adherence, body composition, health-related outcomes, or weight management.
- Context (C): clinical, community, workplace, home-based, sports, and digital settings in which AI technologies were used to individualize lifestyle interventions.

Accordingly, this scoping review mapped and synthesized the available evidence regarding AI-supported personalization across these complementary lifestyle domains, with particular emphasis on their potential role in training optimization and weight management, while identifying current evidence gaps and practical limitations.

## 2. Materials and Methods

This scoping review was conducted in accordance with the Preferred Reporting Items for Systematic Reviews Extension for Scoping Reviews (PRISMA-ScR) guidelines [20]. To ensure methodological transparency and reduce the risk of reporting bias, the review protocol was retrospectively registered on the Open Science Framework (OSF) on 9 June 2026 (https://doi.org/10.17605/OSF.IO/8Z9XW). The eligibility criteria were developed in accordance with the PCC framework [19]. Although the included studies addressed different application domains, such as physical activity promotion, nutrition guidance, musculoskeletal rehabilitation, and behavioral engagement, these were considered complementary components of personalized training and weight-management interventions because they represent lifestyle domains in which AI is used to individualize recommendations according to users’ characteristics, preferences, health status, and behavioral responses.

### 2.1. Search Strategy

This scoping review followed the PRISMA Extension for Scoping Reviews (PRISMA-ScR) guidelines. Two independent reviewers (NC and AC) systematically searched the Scopus, Web of Science, PubMed and IEEE Xplore databases. The literature search was conducted between 1 April and 20 April 2026, with the final search update performed on 20 April 2026. Database-specific search strategies consisted of combinations of free-text keywords and, where applicable, controlled vocabulary terms related to artificial intelligence, exercise, personalization, and weight-related outcomes. The complete search strategies for each database are provided in the Supplementary File S1. Additional relevant studies were identified through manual screening of the reference lists of eligible articles.

### 2.2. Study Selection

Studies were considered eligible if they evaluated interventions incorporating an artificial intelligence (AI) component used to personalize or adapt recommendations for individual users. AI approaches included machine learning, deep learning, natural language processing, conversational agents, or other AI-based approaches used to personalize exercise prescription, physical activity, nutrition guidance, musculoskeletal rehabilitation, behavioral support, or weight-management recommendations according to individual user characteristics, health status, preferences, or behavioral data. Personalization referred to the adaptation of recommendations based on user-specific information rather than the delivery of standardized content. Eligible studies included randomized controlled trials, pilot studies, observational studies, qualitative studies, and comparative evaluations investigating AI-supported personalized interventions related to exercise, physical activity, nutrition, rehabilitation, or weight management. Reviews, editorials, conference abstracts, protocols, conceptual papers, and other publications without original empirical data were excluded. Eligibility criteria were established a priori to include English-language studies published from 1 January 2017 onward in order to capture contemporary developments in AI-supported health interventions. After importing records into Zotero and removing duplicates, four independent reviewers (AC, BK, MS, and FM) independently screened titles and abstracts and subsequently assessed full-text articles for eligibility according to the predefined criteria. Disagreements were resolved through discussion until consensus was reached. When consensus could not be achieved, a senior reviewer (NC) was consulted to make the final decision. Studies were excluded if they did not evaluate an AI-supported personalized intervention relevant to the review objectives, did not report behavioral, physical, nutritional, rehabilitation, or weight-related outcomes, or did not contain original empirical data. Because this was a scoping review, no formal risk-of-bias or methodological quality assessment was performed, as the aim was to map and summarize the available evidence rather than critically appraise individual studies.

### 2.3. Data Extraction and Synthesis

Data from the included studies were extracted by two reviewers (AC and MS) using a standardized data-charting form developed for this scoping review in a Microsoft Excel spreadsheet. Two additional reviewers (FM and BK) independently verified the extracted data against the original articles. Extracted information included study characteristics (authors, year of publication, country, and study design), participant characteristics (sample size, age, sex, and population characteristics), artificial intelligence technologies used (e.g., conversational AI, machine learning, reinforcement learning, deep learning, and large language models), intervention characteristics (duration, delivery platform, and application area), outcomes assessed (physical activity, weight-related outcomes, adherence, engagement, behavioral outcomes, fitness measures, nutritional outcomes, and health-related outcomes), and key findings.

Any discrepancies identified during data extraction or verification were resolved through discussion and consensus among the reviewers. When consensus could not be reached, a senior reviewer (NC) was consulted to make the final decision. Given the exploratory nature of a scoping review and the heterogeneity of study designs, AI technologies, intervention protocols, and outcome measures, no formal meta-analysis was conducted. Instead, findings were synthesized descriptively and organized into thematic categories, including AI approaches, application areas, target populations, outcomes measured, key trends, and research gaps.

## 3. Results

### 3.1. Studies Identification

A total of 611 records were identified through database searching, including 279 records from PubMed, 293 from Web of Science, 22 from Scopus, and 17 from IEEE Xplore. After removing 206 duplicate records, 405 unique records remained for title and abstract screening. At this stage, 348 records were excluded because they did not meet the predefined eligibility criteria, leaving 57 reports for retrieval and full-text assessment.

**Figure 1:**
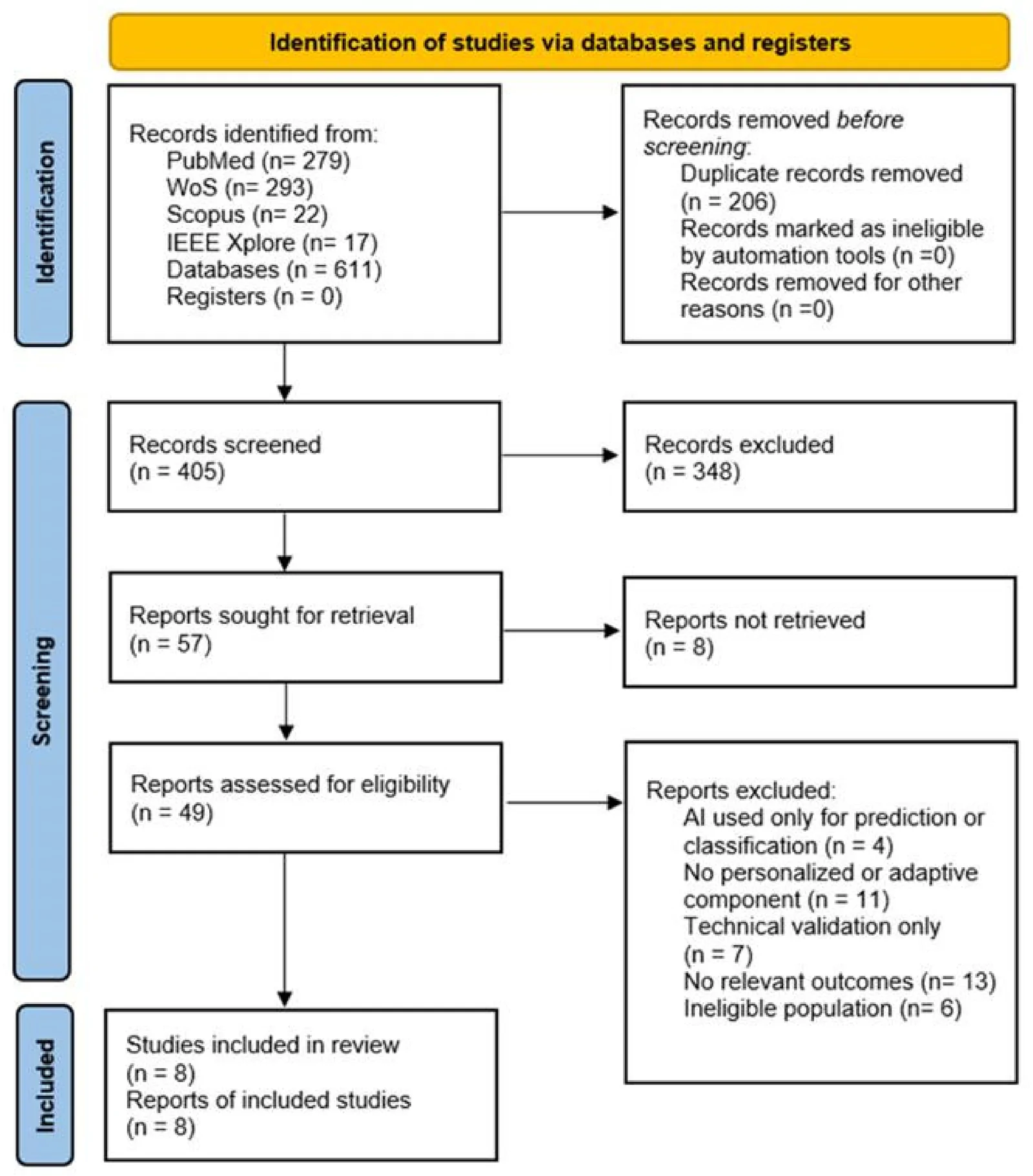
PRISMA-ScR flow diagram illustrating the process of study identification, screening, eligibility assessment, and inclusion.

Of the 57 reports sought for retrieval, eight could not be obtained, and 49 full-text reports were assessed for eligibility. Following full-text evaluation, 41 reports were excluded. The reasons for exclusion were the use of artificial intelligence exclusively for prediction or classification without delivering a personalized intervention (n = 4), the absence of a personalized or adaptive component (n = 11), technical validation without evaluation of relevant intervention outcomes (n = 7), the absence of relevant physical activity, exercise, nutritional, rehabilitation, behavioral, or weight-management outcomes (n = 13), and an ineligible study population (n = 6). Consequently, eight studies met all eligibility criteria and were included in the final scoping review. The study-selection process was reported in accordance with the PRISMA 2020 statement and the PRISMA Extension for Scoping Reviews (PRISMA-ScR) and is presented in Figure 1 [21].

### 3.2. Characteristics of Included Studies

The eight studies included in this scoping review were published between 2017 and 2024 and were conducted in Japan, Australia, the United States, South Korea, and Norway [22,23,24,25,26,27,28,29]. The studies employed heterogeneous methodological designs, including randomized controlled trials, observational and retrospective cohort studies, a single-arm proof-of-concept study, and a cross-sectional qualitative survey. Intervention duration ranged from three weeks to 12 months, while sample sizes varied from 24 participants to 3933 enrolled individuals. The included populations comprised adolescents and adults with overweight or obesity, physically inactive adults, individuals at increased risk of type 2 diabetes, sedentary workers with musculoskeletal symptoms, patients referred for specialist rehabilitation, and healthcare professionals evaluating AI-generated dietary plans [22,23,24,25,26,27,28,29]. The main characteristics of the included studies are summarized in Table 1.

### 3.3. Types of Artificial Intelligence Used

The included studies investigated a variety of artificial intelligence (AI) technologies intended to provide personalized support for physical activity, weight management, dietary modification, and health behavior change. The AI approaches included conversational systems, chatbot-based interventions, machine learning-driven applications, computer vision, knowledge-based systems, and large language models.

Three studies used AI-based conversational coaching systems delivered through digital or mobile platforms. Anan et al. (2021) [22] evaluated an AI-assisted interactive health promotion system (secaide Ver. 0.9), which used a chatbot integrated into the LINE messaging application to provide daily exercise instructions, lifestyle recommendations, reminders, and individualized responses. Maher et al. (2020) [27] investigated “Paola,” a natural language processing (NLP)-based virtual health coach developed using IBM Watson Virtual Assistant, which provided individualized education, goal setting, weekly check-ins, and feedback. Similarly, Stein and Brooks (2017) [25] examined Lark, a fully automated AI health coach based on cognitive behavioral therapy principles that delivered text-based coaching related to weight management and healthy eating.

Machine learning-based adaptive interventions were evaluated by Forman et al. (2019) [23], who investigated the OnTrack smartphone application. The system used ecological momentary assessment (EMA) data and machine learning algorithms to predict dietary lapse risk and deliver personalized just-in-time adaptive interventions (JITAIs). Graham et al. (2022) [24] evaluated an AI-powered conversational coaching system (Lark Diabetes Prevention Program), which provided automated coaching, feedback, self-monitoring support, and guidance based on user interactions and behavioral data.

One study investigated an AI-based exercise application using computer vision technology. Oh et al. (2022) [29] evaluated the SUKIA home exercise application, which incorporated a convolutional neural network (CNN) for movement recognition and provided real-time visual and auditory feedback during exercise sessions.

The application of knowledge-based AI was examined by Marcuzzi et al. (2023) [28], who investigated the SELFBACK application. The system used case-based reasoning to generate individualized self-management recommendations related to physical activity, exercise, education, and behavioral reinforcement for individuals with musculoskeletal conditions.

Finally, Kim et al. (2024) [26] investigated the use of a large language model (LLM), ChatGPT-4.0, to generate general and personalized weight-loss diet plans. The study assessed these plans through evaluations conducted by healthcare professionals, including physicians, registered dietitians, and nurse practitioners.

Overall, the included studies used diverse AI approaches, ranging from conversational and knowledge-based systems to machine learning, natural language processing, computer vision, and large language models. The mechanisms of personalization also varied, including individualized responses, behavioral data, ecological momentary assessment, movement recognition, user interaction, and information provided for dietary-plan generation.

### 3.4. Application Areas

The included studies investigated AI-supported interventions across several domains related to physical activity, weight management, dietary behavior, musculoskeletal self-management, and health behavior modification.

Weight management and obesity-related interventions represented one of the main application areas. Three studies focused primarily on weight-related outcomes. Stein and Brooks (2017) [25] examined an AI health coaching system among adults with overweight or obesity, with outcomes including body weight and meal quality. Forman et al. (2019) [23] investigated an AI-driven adaptive intervention integrated with a commercial weight-loss program and assessed weight loss and dietary lapses. Graham et al. (2022) [24] evaluated an AI-supported diabetes prevention program in adults at increased risk for type 2 diabetes, with outcomes including weight-loss maintenance and achievement of ≥5% weight loss.

AI was also applied to physical activity promotion and exercise delivery. Anan et al. (2021) [22] investigated an AI-assisted health promotion system that provided exercise instructions and lifestyle recommendations among predominantly sedentary workers with musculoskeletal symptoms. Oh et al. (2022) [29] evaluated an AI-based home exercise application that provided interactive exercise guidance and real-time movement feedback among adolescents with overweight or obesity.

Dietary modification and lifestyle behavior were examined in two studies. Maher et al. (2020) [27] investigated an AI virtual health coach in inactive adults who were not meeting physical activity recommendations and were not following a Mediterranean-style diet. Kim et al. (2024) [26] evaluated AI-generated personalized dietary plans through expert assessment rather than through a dietary intervention in participants.

AI applications were also investigated in musculoskeletal rehabilitation and self-management. Marcuzzi et al. (2023) [28] evaluated the SELFBACK application among adults with neck and/or low back pain, assessing musculoskeletal health, pain-related outcomes, self-efficacy, and quality of life.

Taken together, the included studies covered several application areas, with weight management, physical activity, dietary behavior, and musculoskeletal self-management representing the main domains. However, the substantial variation in intervention purpose and target population indicates that the available evidence extends beyond training and weight reduction alone.

### 3.5. Populations

The included studies involved diverse populations and clinical or behavioral contexts. Sample sizes ranged from 24 participants in Oh et al. (2022) [29] to 3933 enrolled participants in the digital diabetes prevention cohort investigated by Graham et al. (2022) [24].

Three studies focused primarily on adults with overweight or obesity or increased metabolic risk. Stein and Brooks (2017) [25] included overweight and obese adults recruited from primary care settings who were active users of an AI-based weight-loss application. Forman et al. (2019) [23] investigated adults seeking weight reduction with overweight or obesity (mean BMI = 34.32 ± 5.65 kg/m^2^). Graham et al. (2022) [24] included adults with BMI ≥ 25 kg/m^2^ who had prediabetes or were at increased risk for type 2 diabetes.

Other studies targeted populations with specific physical activity or health characteristics. Anan et al. (2021) [22] included predominantly sedentary company workers aged 20–64 years with neck, shoulder, or low back symptoms. Maher et al. (2020) [27] recruited inactive community-dwelling adults aged 45–75 years who were not meeting physical activity recommendations and were not following a Mediterranean-style diet. Marcuzzi et al. (2023) [28] included adults referred to specialist rehabilitation services because of neck and/or low back pain.

The application of AI among younger participants was investigated by Oh et al. (2022) [29], which included adolescents aged 10–17 years with overweight or obesity. In contrast, Kim et al. (2024) [26] investigated healthcare professionals, including physicians, registered dietitians, and nurse practitioners specializing in obesity medicine or clinical nutrition, who evaluated AI-generated dietary recommendations.

Overall, the included populations differed substantially in age, health status, clinical characteristics, and intervention objectives. They ranged from inactive but otherwise community-dwelling adults to adolescents with overweight or obesity, adults with metabolic risk, individuals receiving musculoskeletal rehabilitation, and healthcare professionals evaluating AI-generated dietary plans.

### 3.6. Outcomes Measured

The included studies assessed a broad range of outcomes related to body weight, physical activity, dietary behavior, health status, and engagement with AI-supported interventions (Table 2).

Weight-related outcomes were assessed in three studies. Stein and Brooks (2017) [25] measured weight loss, meal quality, user engagement, and satisfaction. Forman et al. (2019) [23] assessed weight loss, dietary lapses, participant satisfaction, application engagement, and algorithm accuracy. Graham et al. (2022) [24] evaluated weight-loss maintenance at 12 months, achievement of ≥5% weight loss, weight nadir, BMI change, and predictors of successful weight loss.

Physical activity and fitness outcomes were assessed in three studies. Maher et al. (2020) [27] measured weekly moderate-to-vigorous physical activity (MVPA) and adherence to a Mediterranean-style diet, together with body weight, waist circumference, blood pressure, and feasibility outcomes. Oh et al. (2022) [29] assessed calorie consumption, VO_2_max, six-minute walk test performance, BMI, perceived exertion, motivation, enjoyment, and perceived exercise effectiveness. Anan et al. (2021) [22] evaluated musculoskeletal symptoms, subjective symptom improvement, and adherence to the intervention.

Behavioral and engagement-related outcomes were also examined. Anan et al. (2021) [22], Maher et al. (2020) [27], Stein and Brooks (2017) [25], and Graham et al. (2022) [24] included measures of adherence, engagement, retention, or interaction with the AI system.

Clinical and patient-reported outcomes were examined by Marcuzzi et al. (2023) [28], including musculoskeletal health, pain-related disability, pain intensity, self-efficacy, quality of life, illness perception, and global perceived effect.

Finally, Kim et al. (2024) [26] evaluated AI-generated dietary plans according to effectiveness, balancedness, comprehensiveness, flexibility, applicability, overall impression, safety, and intention to use. These outcomes represented expert evaluations of AI-generated dietary plans rather than changes in participants’ body weight or dietary behavior.

### 3.7. Key Trends

Several descriptive patterns emerged across the eight included studies. First, five of the eight studies [22,23,24,25,27] incorporated conversational or text-based AI coaching, reminders, feedback, or repeated user interaction. These systems generally provided ongoing digital support rather than one-time information delivery.

Second, four studies [22,24,25,27] reported outcomes related to adherence, engagement, retention, or frequency of interaction. These studies generally reported favorable engagement-related findings; however, their designs differed substantially, including randomized, single-arm, and retrospective observational designs. Therefore, the findings should be interpreted as reported associations or changes rather than as evidence of a consistent causal effect of AI on adherence.

Third, three studies primarily examined weight-related outcomes [23,24,25]. These studies reported changes in body weight or weight-related outcomes, although the magnitude and interpretation of these changes differed across study designs and intervention contexts. Forman et al. (2019) [23] reported greater weight loss in one dietary-program subgroup when OnTrack was combined with Weight Watchers, whereas Stein and Brooks (2017) [25] and Graham et al. (2022) [24] were observational in design and therefore cannot independently establish causal effects.

Fourth, findings were not uniformly favorable across all outcomes. Marcuzzi et al. (2023) [28], a three-arm randomized clinical trial, did not find statistically significant superiority of the AI-based SELFBACK intervention over usual care or e-Help for the primary musculoskeletal outcome at three months, with no significant between-group differences for most secondary outcomes.

Finally, the included studies represented increasingly diverse AI technologies, including conversational AI, NLP, machine learning, computer vision, case-based reasoning, and large language models. This technological heterogeneity, together with variation in populations and outcomes, limits direct comparison across studies.

### 3.8. Research Gaps

Despite the increasing use of AI in personalized health interventions, several research gaps were identified.

First, the evidence base consisted of only eight studies and included heterogeneous study designs. Although four studies used randomized controlled designs, the remaining studies included a single-arm pre-post study, a retrospective observational study, a retrospective cohort study, and a cross-sectional expert survey. Consequently, the evidence does not provide a uniform basis for determining intervention effectiveness.

Second, intervention duration and follow-up varied considerably, and several studies involved relatively short intervention periods. Longer-term evidence regarding maintenance of weight loss, physical activity behavior, and dietary changes remains limited.

Third, considerable heterogeneity was observed in AI technologies, personalization mechanisms, target populations, intervention protocols, and outcome measures. This variability makes direct comparisons between studies difficult and prevents meaningful aggregation of findings across the included evidence.

Fourth, the degree and mechanism of personalization were not consistently described across studies. Some systems used behavioral or ecological momentary assessment data to adapt recommendations, whereas others provided individualized responses or movement-based feedback. Future research should explicitly report which participant data are used for personalization, how recommendations are adapted, and whether adaptation occurs continuously or only at predefined stages.

Fifth, limited evidence is available regarding the integration of AI systems with professional healthcare or exercise support. Future studies should investigate hybrid models combining AI-supported monitoring and feedback with supervision from exercise professionals, physicians, dietitians, or other qualified practitioners.

Sixth, methodological limitations should be considered when interpreting the findings. A formal risk-of-bias or methodological quality assessment was not conducted in this scoping review. Therefore, the findings should be interpreted as a mapping of the available evidence rather than as an assessment of the certainty or effectiveness of AI interventions.

Seventh, the evidence base may be affected by the databases searched and the language restrictions applied during study identification. The selection of databases and restriction to English-language publications may have resulted in relevant evidence being omitted. Future reviews should consider broader database coverage and inclusion of studies published in additional languages where feasible.

Finally, issues related to equitable access to AI-supported interventions remain insufficiently investigated. Digital literacy, socioeconomic circumstances, access to smartphones and reliable internet services, and users’ ability to engage with AI technologies may influence who can benefit from such interventions. Future research should therefore examine accessibility, digital literacy, affordability, and potential disparities in access alongside intervention outcomes.

## 4. Discussion

### 4.1. AI-Supported Physical Activity Monitoring and Exercise Delivery

The included studies examined AI technologies as potential tools for supporting physical activity, exercise delivery, and behavioral monitoring. The included studies used AI systems to provide exercise instructions, reminders, movement recognition, feedback, and repeated digital interaction. However, the available evidence differs substantially in study design, population, and intervention characteristics, limiting conclusions regarding the effectiveness of AI-supported exercise interventions as a group.

Anan et al. (2021) [22] reported greater reductions in neck/shoulder and low back symptoms in the AI-assisted intervention group compared with the control group after 12 weeks, together with a high reported adherence rate. The intervention provided daily exercise instructions and lifestyle information through a chatbot. These findings suggest that the intervention was associated with favorable changes in symptoms and adherence within the study population, although the unblinded design and nature of the intervention should be considered when interpreting the findings.

Oh et al. (2022) [29] compared an AI-based home exercise application incorporating CNN-based movement recognition with a Nintendo Switch-based intervention. Both interventions were associated with improvements in BMI, VO_2_max, and six-minute walk test performance, while the SUKIA intervention showed greater changes in some outcomes. The use of real-time movement recognition and feedback represents a distinct technological approach because the system responded to participants’ exercise movements rather than relying solely on text-based coaching.

Nevertheless, these findings do not establish that AI-supported exercise delivery can replace professional supervision. Exercise prescription may require consideration of injury history, technical ability, physical limitations, and changes in individual condition. Therefore, the current evidence is better interpreted as indicating potential applications of AI as a supplementary tool for exercise support and monitoring.

### 4.2. Personalization and Adaptive Coaching in AI-Based Interventions

Personalization was a common characteristic of the included AI interventions, although the mechanisms used to achieve personalization differed between studies. Some systems relied on behavioral data and repeated interactions, whereas others used ecological momentary assessment, movement recognition, or information provided by users.

Forman et al. (2019) [23] provide a clear example of adaptive personalization. The OnTrack application used ecological momentary assessment data and machine learning to predict dietary lapse risk and provide just-in-time interventions. The study reported greater weight loss in the OnTrack + Weight Watchers group within the BTS dietary program compared with Weight Watchers alone. The algorithm also demonstrated measurable sensitivity and specificity in predicting dietary lapses. These findings illustrate how behavioral data can be incorporated into an adaptive intervention, although the observed effects should be considered within the specific study and dietary-program context.

Graham et al. (2022) [24] also reported an association between the frequency of AI coaching exchanges and weigh-ins and the likelihood of achieving ≥ 5% weight loss. However, because this study used a retrospective cohort design, the association cannot establish that more frequent AI interactions caused greater weight loss. Higher engagement may itself reflect characteristics of participants who were more likely to achieve weight loss.

Kim et al. (2024) [26] investigated a different form of personalization using ChatGPT-4.0 to generate dietary plans. Healthcare professionals evaluated the resulting plans across several dimensions, and no significant differences were identified between AI-generated and human-created plans across the assessed categories. However, this study evaluated the perceived characteristics of generated plans rather than actual weight-loss outcomes in participants. The reported limitations concerning specificity, portion sizing, affordability, and conflicting dietary considerations further indicate the importance of professional review.

Taken together, these studies illustrate several possible approaches to AI-supported personalization, but they do not establish that one personalization mechanism is superior to another. Future research should provide more detailed descriptions of the data used by algorithms, adaptation procedures, frequency of adaptation, and the extent to which recommendations change in response to individual characteristics.

### 4.3. Behavioral Engagement and Adherence

Four of the eight included studies assessed engagement, adherence, retention, or interaction with AI-supported interventions [22,24,25,27]. These studies generally reported favorable engagement-related findings, although the evidence came from different study designs and should therefore not be interpreted as demonstrating a uniform causal effect.

Maher et al. (2020) [27] reported 90% retention after a 12-week intervention using the Paola virtual health coach, together with changes in physical activity and Mediterranean diet adherence. As this was a single-arm pre-post study, the observed changes cannot be attributed exclusively to the AI intervention.

Anan et al. (2021) [22] reported a 92% adherence rate in participants receiving daily chatbot-based exercise and lifestyle guidance. Stein and Brooks (2017) [25] reported that greater engagement with the Lark AI health coach was associated with greater weight loss. Similarly, Graham et al. (2022) [24] identified an association between more frequent AI coaching exchanges and weigh-ins and the likelihood of achieving ≥ 5% weight loss.

These findings suggest that repeated interaction and self-monitoring were common features of the interventions reporting favorable engagement-related outcomes. However, engagement may also reflect participant characteristics, motivation, or baseline readiness to change. Consequently, future controlled studies should examine whether increasing AI interaction independently improves adherence and whether engagement can be maintained over longer periods.

### 4.4. Weight-Related and Health Outcomes

Three of the eight included studies primarily examined weight-related outcomes [23,24,25]. The studies reported changes or associations in weight-related outcomes, although the direction, magnitude, and interpretation of these findings differed substantially across study designs and analytical approaches.

Stein and Brooks (2017) [25], a retrospective observational study, reported a mean weight loss of 2.4% after 15 weeks and an association between greater engagement with the AI health coach and greater weight loss. Because of the observational design, the findings cannot establish a causal relationship between AI coaching and weight loss.

Forman et al. (2019) [23] conducted a randomized controlled trial and reported greater weight loss in the OnTrack + Weight Watchers group than in the Weight Watchers-only group among participants following the BTS diet (4.7% vs. 2.6%). This finding suggests a between-group difference within the specific trial context, although the reported effect differed according to the dietary program.

Graham et al. (2022) [24] reported greater weight-loss maintenance among CDC qualifiers compared with non-qualifiers and identified an association between AI coaching interactions, weigh-ins, and achievement of ≥5% weight loss. However, the retrospective cohort design limits causal interpretation.

The findings were not uniformly favorable across all health-related applications. Marcuzzi et al. (2023) [28] did not find statistically significant superiority of the AI-based SELFBACK intervention over usual care or e-Help for the primary musculoskeletal health outcome at three months. This finding is important because it demonstrates that the presence of an AI component does not necessarily result in superior outcomes compared with conventional or digital care.

Overall, the available studies report changes or between-group differences in several weight-related and health outcomes, but the heterogeneity of designs and populations, together with the absence of formal risk-of-bias assessment, prevents firm conclusions regarding the overall effectiveness of AI-supported interventions.

### 4.5. Practical Limitations and Future Perspectives

Several practical and methodological limitations should be considered when interpreting the available evidence.

First, the number of included studies was small, with only eight studies meeting the eligibility criteria. The evidence also included randomized controlled trials, observational studies, a single-arm intervention, and an expert survey. This methodological heterogeneity limits direct comparison and prevents a uniform assessment of intervention effectiveness.

Second, a formal risk-of-bias or methodological quality assessment was not conducted. Consequently, the review maps reported findings but does not establish the certainty of the evidence or determine whether observed differences were affected by systematic sources of bias. Future evidence syntheses and primary studies should incorporate appropriate methodological appraisal where the review objective requires assessment of intervention effectiveness.

Third, intervention duration and follow-up were often limited. Longer-term studies are needed to determine whether changes in physical activity, dietary behavior, engagement, and weight can be maintained over extended periods.

Fourth, AI technologies differed substantially across the included studies. Chatbots, NLP systems, machine learning algorithms, computer vision, case-based reasoning, and large language models were used for different purposes. Future studies should report personalization mechanisms in greater detail, including the participant information used by the AI system, the algorithmic process used to adapt recommendations, and the frequency of adaptation.

Fifth, the role of professional supervision remains important. Kim et al. (2024) [26], for example, identified limitations in AI-generated dietary plans related to specificity, portion sizing, affordability, and conflicting dietary considerations. These findings highlight the potential importance of appropriate human oversight when AI-generated recommendations involve health or nutrition decisions.

Sixth, the evidence may be influenced by the databases, language, and access restrictions used during study identification. The selection of databases and restriction to English-language publications may have limited the range of evidence captured by the review. In addition, the PubMed search was restricted to “Free full text,” while the Web of Science search was restricted to “Open Access.” These filters may have introduced availability bias by excluding potentially eligible studies solely on the basis of their accessibility rather than their methodological or topical relevance. This limitation is particularly important in the context of a scoping review, which aims to comprehensively map the available evidence within a given field. Consequently, the use of access-based filters may have reduced the comprehensiveness of the evidence identified and should be considered when interpreting the findings of this review. Future reviews should avoid access-based restrictions where possible and consider broader database coverage and multilingual searches to ensure a more comprehensive representation of the available literature. Finally, equitable access represents an important consideration for future implementation. AI-supported interventions may require smartphones, internet connectivity, digital literacy, and the ability to interact effectively with automated systems. Socioeconomic barriers, differences in digital literacy, affordability, and accessibility may therefore influence who can use and benefit from AI-supported interventions. These issues should be incorporated into future evaluations of AI-based health and exercise technologies.

Future research should prioritize adequately powered randomized controlled trials with longer follow-up, standardized outcome measures, transparent descriptions of AI personalization mechanisms, and direct comparisons between different AI approaches. Research should also investigate hybrid models combining AI-supported monitoring and feedback with professional supervision, particularly for individuals with complex clinical or behavioral needs.

## 5. Conclusions

This scoping review mapped the available evidence concerning the use of artificial intelligence (AI) for personalized training, weight management, dietary modification, and related health behaviors. The eight included studies investigated a diverse range of technologies, including conversational chatbots, natural language processing systems, machine learning algorithms, computer vision applications, knowledge-based systems, and large language models.

The included studies reported a range of findings related to physical activity, dietary behavior, engagement, musculoskeletal outcomes, and weight-related measures. Several studies reported favorable changes or between-group differences in selected outcomes, while one randomized clinical trial did not demonstrate statistically significant superiority of an AI-based intervention over comparator conditions. The findings therefore indicate potential applications of AI-supported personalization but do not establish consistent intervention effectiveness across the available evidence.

Interpretation is limited by the small number of included studies, substantial heterogeneity in study designs, populations, AI technologies, intervention protocols, and outcome measures, as well as the absence of a formal risk-of-bias or methodological quality assessment. In addition, several included studies were observational or single-arm in design, limiting causal interpretation of reported changes and associations. The study evaluating AI-generated dietary plans also assessed expert perceptions rather than actual weight-loss outcomes.

The available evidence suggests that AI-supported systems can be used to deliver individualized recommendations, repeated feedback, self-monitoring support, and adaptive interaction across different health and lifestyle contexts; however, the effectiveness of these approaches remains uncertain. However, AI should currently be considered a complementary technology rather than a replacement for professional exercise, healthcare, or nutrition expertise, particularly when interventions involve complex clinical or dietary decisions.

Future research should prioritize larger randomized controlled trials with longer follow-up periods, standardized outcome measures, transparent reporting of personalization mechanisms, and appropriate methodological appraisal. Greater attention should also be given to algorithm transparency, data privacy, digital literacy, affordability, equitable access, and the integration of AI with professional supervision. Such research would help clarify the circumstances under which AI-supported personalization may contribute to sustained changes in physical activity, dietary behavior, and weight management.

## Figures and Tables

**Table 1:** Characteristics of the studies included in the review.

Study	Country	Study Design	Intervention Duration	Sample Size	Age	Gender	Sample Characteristics
Anan et al. (2021) [22]	Japan	Two-armed randomized controlled trial (RCT), unblinded	12 weeks	94 participants (48 intervention, 46 control)	41.8 ± 8.7 years (intervention); 42.4 ± 8.0 years (control)	72 male 22 female	Company workers aged 20–64 years with neck/shoulder pain/stiffness and/or low back pain; mostly sedentary white-collar employees and engineers
Maher et al. (2020) [27]	Australia	Single-arm pre-post proof-of-concept study	12 weeks	31 participants	56.2 ± 8.0 years	10 male 21 female	Inactive community-dwelling adults aged 45–75 years who were not meeting physical activity guidelines and were not following a Mediterranean-style diet; most participants were overweight or obese.
Stein & Brooks (2017) [25]	USA	Longitudinal observational (retrospective) study	15 weeks	70	46.9	74.5% female (35/47 with available gender data)	Overweight and obese adults (BMI ≥ 25 kg/m^2^) recruited from six primary care practices; active users of the Lark Weight Loss AI app
Oh et al. (2022) [29]	South Korea	Randomized controlled trial (RCT)	3 weeks	24	13.2 ± 3.6	20 male 4 female	Adolescents aged 10–17 years with overweight/obesity (BMI > 85th percentile), no pharmacological treatment or major comorbidities
Forman et al. (2019) [23]	USA	Randomized Controlled Trial (RCT), parallel-group	10 weeks	N = 181 (185 randomized; 181 analyzed)	Mean age = 46.29 ± 13.58 years	85.1% female	Adults with overweight or obesity (mean BMI = 34.32 ± 5.65 kg/m^2^), seeking weight loss; BMI 25–50 kg/m^2^; iPhone owners living in the USA. Participants were randomized to the Weight Watchers (WW) digital program alone or WW + OnTrack (JITAI smartphone application). Sample was 73.5% White; excluded individuals with recent ≥5% weight loss, bariatric surgery, pregnancy, eating disorders, recent medication changes affecting weight, or serious medical conditions influencing weight/appetite.
Marcuzzi A. et al. (2023) [28]	Norway	Randomized Clinical Trial (3 parallel groups)	6 months (primary outcome at 3 months)	294 (99 app, 98 e-Help, 97 usual care)	50.6 ± 14.9	121 male 173 female	Adults (≥18 years) with neck and/or low back pain referred to specialist outpatient rehabilitation care.
Graham et al. (2022) [24]	USA	Retrospective longitudinal cohort study	12 months	3933 enrolled; primary analysis n = 414; secondary analysis n = 3148	53.2 ± 0.5 years	273 women (66%), 141 men (34%)	Adults (>18 years) with prediabetes or high risk for type 2 diabetes, BMI ≥ 25 kg/m^2^, enrolled in a CDC-recognized digital Diabetes Prevention Program.
Kim et al. (2024) [26]	USA	Cross-sectional qualitative survey study	Not applicable (survey conducted Apr–May 2023)	95 invited; 67 completed survey; 57 evaluated personalized AI diet plan	Not reported (median professional experience: 9 years)	Not reported	Physicians, registered dietitians, and nurse practitioners specializing in obesity medicine or clinical nutrition.

Legend: Abbreviations: AI, artificial intelligence; RCT, randomized controlled trial; BMI, body mass index; WW, Weight Watchers; JITAI, just-in-time adaptive intervention; CDC, Centers for Disease Control and Prevention; SD, standard deviation; n, number of participants.

**Table 2:** Intervention design and outcome measures in the studies included in the review.

Study	Outcomes	Main Findings	AI Technology and Personalization Mechanism
Anan et al. (2021) [22]	Weight loss, meal quality, user engagement, and user satisfaction.	Mean weight loss was 2.4% (2.4 kg) after 15 weeks, while the proportion of healthy meals increased by 31%. Greater engagement with the AI-based intervention was associated with greater weight loss. High levels of user satisfaction were also reported.	An AI-assisted interactive health-promotion system (secaide Ver. 0.9) delivered daily 1 min exercise instructions and lifestyle information via a chatbot integrated into the LINE mobile messaging application. The system provided reminders, individualized responses, motivational messages, and engagement monitoring.
Maher et al. (2020) [27]	Calorie consumption, VO_2_max, 6 min walk test (6MWT), body mass index (BMI), Rating of Perceived Exertion (RPE), motivation, enjoyment, and perceived exercise effectiveness.	Both interventions were associated with improvements in BMI, VO_2_max, and 6MWT performance. Compared with the Nintendo Switch intervention, SUKIA showed greater improvements in calorie consumption and VO_2_max, as well as greater changes in RPE. Both interventions were well accepted by the participants.	The intervention used “Paola,” a natural language processing (NLP)-based virtual health coach developed using IBM Watson Virtual Assistant and delivered through Slack (Slack Technologies; version not specified in the original study). Paola provided personalized education, goal setting, weekly check-ins, feedback, and 24/7 responses to participants’ questions. .
Stein & Brooks (2017) [25]	Weight loss, dietary lapses, participant satisfaction, app engagement, and algorithm accuracy.	The OnTrack + WW group showed greater weight loss than the WW-only group among participants following the BTS diet (4.7% vs. 2.6%). Dietary lapses decreased over time. The algorithm demonstrated 69.2% sensitivity and 83.8% specificity for predicting dietary lapse risk.	Lark HCAI, a fully automated, text-based AI health coach using cognitive behavioral therapy (CBT) principles. The system provided personalized, 24/7 text-based coaching related to weight management and healthy eating.
Oh et al. (2022) [29]	Musculoskeletal Health Questionnaire (MSK-HQ; primary outcome); pain-related disability; pain intensity; self-efficacy; quality of life; illness perception; and global perceived effect.	The AI-based app used as an adjunct to usual care did not show statistically significant superiority over usual care alone or e-Help in improving musculoskeletal health at 3 months. No significant between-group differences were observed for most secondary outcomes.	SUKIA, an AI-based home-exercise application using a convolutional neural network (CNN) for gesture recognition, provided real-time visual and auditory feedback during exercise. The system enabled interactive home-based exercise with individualized feedback based on users’ movements.
Forman et al. (2019) [23]	Percentage of weight loss maintained at 12 months; achievement of ≥5% weight loss; weight nadir; change in BMI; and predictors of successful weight loss.	CDC qualifiers maintained significantly greater weight loss at 12 months than non-qualifiers (5.3% vs. 3.3%; *p* = 0.015). Forty percent of CDC qualifiers achieved ≥5% weight loss. More frequent AI coaching exchanges and weigh-ins were independently associated with a greater likelihood of achieving ≥ 5% weight loss.	A machine learning-based just-in-time adaptive intervention (JITAI) smartphone application that used ecological momentary assessment (EMA) data to predict the risk of dietary lapses and deliver personalized, real-time interventions.
Marcuzzi A. et al. (2023) [28]	Effectiveness, balancedness, comprehensiveness, flexibility, applicability, overall impression, safety, and intention to use personalized AI-generated diet plans.	No significant differences were found between AI-generated and human-created diet plans across the evaluated categories. A total of 79.1% of experts were unable to distinguish the AI-generated diet plan from the human-created plan. Personalized AI-generated diet plans received above-neutral ratings across all evaluation variables.	The SELFBACK app used a knowledge-based AI approach incorporating case-based reasoning to provide individualized self-management recommendations. The system provided recommendations related to exercise, physical activity, education, and behavioral reinforcement based on user information.
Graham et al. (2022) [24]	Weight loss, meal quality, user engagement, and user satisfaction.	Mean weight loss was 2.4% (2.4 kg) after 15 weeks, while the proportion of healthy meals increased by 31%. Greater engagement with the AI-based intervention was associated with greater weight loss. High levels of user satisfaction were also reported.	Lark DPP, a conversational AI-powered mobile application, delivered personalized, real-time, 24/7 coaching and feedback based on the CDC PreventT2 curriculum. The application supported self-monitoring through coaching interactions and weight tracking.
Kim et al. (2024) [26]	Calorie consumption, VO_2_max, 6 min walk test (6MWT), body mass index (BMI), Rating of Perceived Exertion (RPE), motivation, enjoyment, and perceived exercise effectiveness.	Both interventions were associated with improvements in BMI, VO_2_max, and 6MWT performance. Compared with the Nintendo Switch intervention, SUKIA showed greater improvements in calorie consumption and VO_2_max, as well as greater changes in RPE. Both interventions were well accepted by the participants.	ChatGPT 4.0, a large language model (LLM), was used to generate general and personalized weight-loss diet plans. The AI-generated plans were evaluated by physicians, registered dietitians, and nurse practitioners. Reported limitations included insufficient specificity, affordability considerations, portion sizing, and conflicting dietary considerations, indicating the need for expert review.

Legend: AI = artificial intelligence; MVPA = moderate-to-vigorous physical activity; NLP = natural language processing; CBT = cognitive behavioral therapy; HCAI = Health Coach Artificial Intelligence; VO_2_max = maximal oxygen uptake; 6MWT = six-minute walk test; BMI = body mass index; RPE = Rating of Perceived Exertion; CNN = convolutional neural network; JITAI = just-in-time adaptive intervention; EMA = ecological momentary assessment; WW = Weight Watchers; BTS = Before the Scale (diet program); MSK-HQ = Musculoskeletal Health Questionnaire; CDC = Centers for Disease Control and Prevention; DPP = Diabetes Prevention Program.

## Data Availability

The data supporting the findings of this study are available within the article and its Supplementary Materials. No additional datasets were generated or analyzed beyond those included in the reviewed literature.

## Supplementary Materials

The following supporting information can be downloaded at: https://www.mdpi.com/article/10.3390/jfmk11030347/s1, Supplementary File S1. Complete search strategies, search strings, and filters applied for PubMed, Web of Science, Scopus, and IEEE Xplore.
