# Supplementary material for: Personalization of Training and Weight Reduction Using Artificial Intelligence: A Scoping Review of Current Evidence and Practical Limitations
Source: J Funct Morphol Kinesiol. 2026 Aug 31;11(3):347. doi: 10.3390/jfmk11030347 (PMC13608367; doi:10.3390/jfmk11030347)
Supplement: Supplementary file 1 [file jfmk-11-00347-s001.zip › jfmk-4424456-supplementary.pdf]

Supplementary File S1

The complete search strings for each database are presented below.

machine learning, deep learning, chatbot, conversational agent, reinforcement, learning large language model, obesity, weight management, nutrition, diet, exercise prescription, personalized intervention.

PubMed- 279

Search String: ("Artificial Intelligence"[Mesh] OR "artificial intelligence"[Title/Abstract] OR "machine learning"[Title/Abstract] OR "deep learning"[Title/Abstract] OR "reinforcement learning"[Title/Abstract] OR "large language model"[Title/Abstract] OR "large language models"[Title/Abstract] OR "generative artificial intelligence"[Title/Abstract] OR "generative AI"[Title/Abstract] OR ChatGPT[Title/Abstract] OR chatbot*[Title/Abstract] OR "conversational agent"[Title/Abstract] OR "conversational agents"[Title/Abstract] OR "virtual coach"[Title/Abstract] OR "virtual coaches"[Title/Abstract] OR "digital coach"[Title/Abstract] OR "digital coaches"[Title/Abstract] OR "intelligent coaching system"[Title/Abstract] OR "intelligent coaching systems"[Title/Abstract] OR "recommendation system"[Title/Abstract] OR "recommendation systems"[Title/Abstract] OR "recommender system"[Title/Abstract] OR "recommender systems"[Title/Abstract]) AND ("Exercise"[Mesh] OR "Exercise Therapy"[Mesh] OR "Motor Activity"[Mesh] OR "Weight Loss"[Mesh] OR "Weight Management"[Mesh] OR "Obesity"[Mesh] OR "Diet Therapy"[Mesh] OR exercise[Title/Abstract] OR "physical activity"[Title/Abstract] OR "physical activities"[Title/Abstract] OR "exercise prescription"[Title/Abstract] OR "exercise program"[Title/Abstract] OR "exercise programmes"[Title/Abstract] OR "training program"[Title/Abstract] OR "training programmes"[Title/Abstract] OR "physical rehabilitation"[Title/Abstract] OR "exercise rehabilitation"[Title/Abstract] OR "lifestyle intervention"[Title/Abstract] OR "lifestyle interventions"[Title/Abstract] OR "nutritional intervention"[Title/Abstract] OR "nutritional interventions"[Title/Abstract] OR "dietary intervention"[Title/Abstract] OR "dietary interventions"[Title/Abstract] OR obesity[Title/Abstract] OR overweight[Title/Abstract] OR "weight loss"[Title/Abstract] OR "weight reduction"[Title/Abstract] OR "weight management"[Title/Abstract] OR "body composition"[Title/Abstract]) AND ("personalized intervention"[Title/Abstract] OR "personalized interventions"[Title/Abstract] OR "personalised intervention"[Title/Abstract] OR "personalised interventions"[Title/Abstract] OR "individualized intervention"[Title/Abstract] OR "individualized interventions"[Title/Abstract] OR "individualised intervention"[Title/Abstract] OR "individualised interventions"[Title/Abstract] OR "tailored intervention"[Title/Abstract] OR "tailored interventions"[Title/Abstract] OR "adaptive intervention"[Title/Abstract] OR "adaptive interventions"[Title/Abstract] OR "personalized recommendation"[Title/Abstract] OR "personalized recommendations"[Title/Abstract] OR "personalised recommendation"[Title/Abstract] OR "personalised recommendations"[Title/Abstract] OR "individualized recommendation"[Title/Abstract] OR "individualized recommendations"[Title/Abstract] OR "individualised recommendation"[Title/Abstract] OR "individualised recommendations"[Title/Abstract] OR "tailored recommendation"[Title/Abstract] OR "tailored recommendations"[Title/Abstract] OR "personalized feedback"[Title/Abstract] OR "personalised feedback"[Title/Abstract] OR "individualized feedback"[Title/Abstract] OR

"individualised feedback"[Title/Abstract] OR "personalized coaching"[Title/Abstract] OR "personalised coaching"[Title/Abstract] OR "digital coaching"[Title/Abstract] OR "precision nutrition"[Title/Abstract] OR "precision exercise"[Title/Abstract] OR "just-in-time adaptive intervention"[Title/Abstract] OR "just-in-time adaptive interventions"[Title/Abstract] OR JITAI[Title/Abstract])

Filters applied – Free full text, English, Exclude preprints 2017- 2026

Web of Science (n = 293)

Search String:(("artificial intelligence" OR "machine learning" OR "deep learning" OR "reinforcement learning" OR "large language model*" OR "generative artificial intelligence" OR "generative AI" OR ChatGPT OR chatbot* OR "conversational agent*" OR "virtual coach*" OR "digital coach*" OR "intelligent coaching system*" OR "recommendation system*" OR "recommender system*") AND (exercise OR "physical activit*" OR "exercise prescription" OR "exercise program*" OR "exercise programme*" OR "training program*" OR "training programme*" OR "physical rehabilitation" OR "exercise rehabilitation" OR "lifestyle intervention*" OR "nutritional intervention*" OR "dietary intervention*" OR obesity OR overweight OR "weight loss" OR "weight reduction" OR "weight management" OR "body composition") AND ("personalized intervention*" OR "personalised intervention*" OR "individualized intervention*" OR "individualised intervention*" OR "tailored intervention*" OR "adaptive intervention*" OR "personalized recommendation*" OR "personalised recommendation*" OR "individualized recommendation*" OR "individualised recommendation*" OR "tailored recommendation*" OR "personalized feedback" OR "personalised feedback" OR "individualized feedback" OR "individualised feedback" OR "personalized coaching" OR "personalised coaching" OR "digital coaching" OR "precision nutrition" OR "precision exercise" OR "just-in-time adaptive intervention*" OR JITAI))

Filters applied – 2017- 2026, Article, English, Open Accsess

Scopus – 22

Search string :(("artificial intelligence" OR "machine learning" OR "deep learning" OR "reinforcement learning" OR "large language model*" OR "generative artificial intelligence" OR "generative AI" OR ChatGPT OR chatbot* OR "conversational agent*" OR "virtual coach*" OR "digital coach*" OR "intelligent coaching system*" OR "recommendation system*" OR "recommender system*") AND (exercise OR "physical activit*" OR "exercise prescription" OR "exercise program*" OR "exercise programme*" OR "training program*" OR "training programme*" OR "physical rehabilitation" OR "exercise rehabilitation" OR "lifestyle intervention*" OR "nutritional intervention*" OR "dietary intervention*" OR obesity OR overweight OR "weight loss" OR "weight reduction" OR "weight management" OR "body composition") AND ((personali* OR individuali* OR tailor* OR adaptiv*) W/5 (intervention* OR recommend* OR feedback OR coach* OR prescription* OR program* OR programme* OR plan* OR guidance)))

Filters applied – 2017- 2026, English

IEEE Xplore – 17

Search String: ("artificial intelligence" OR "machine learning" OR "deep learning" OR "reinforcement learning" OR "large language model" OR "generative AI" OR ChatGPT OR

chatbot OR "conversational agent" OR "virtual coach" OR "digital coach" OR "recommendation system" OR "recommender system") AND (exercise OR "physical activity" OR "exercise prescription" OR "exercise program" OR "training program" OR rehabilitation OR "lifestyle intervention" OR "nutritional intervention" OR "dietary intervention" OR obesity OR overweight OR "weight loss" OR "weight management" OR "body composition") AND ("personalized intervention" OR "personalised intervention" OR "individualized intervention" OR "individualised intervention" OR "tailored intervention" OR "adaptive intervention" OR "personalized recommendation" OR "personalised recommendation" OR "individualized recommendation" OR "individualised recommendation" OR "tailored recommendation" OR "personalized feedback" OR "personalised feedback" OR "personalized coaching" OR "personalised coaching" OR "digital coaching" OR "precision nutrition" OR "precision exercise" OR "just-in-time adaptive intervention" OR JITAI)

Filters applied – 2017- 2026
